# Recovery from kidney failure associated with chronic thromboembolic pulmonary hypertension following pulmonary thomboendarterectomy

**DOI:** 10.1093/ckj/sfae047

**Published:** 2024-02-28

**Authors:** George M Nassar, Robert Jameson, Steffi Sathiyaraj, Nayda Bidikian, Nelson Villasmil Hernandez, Sandeep Sahay

**Affiliations:** Houston Methodist Hospital – Department of Internal Medicine, Houston, TX, USA; Weill Cornell – Medical College – Department of Internal Medicine, New York, NY, USA; Panoramic Health, a Management Service Organization, Tempe, Arizona, USA; Houston Methodist Hospital – Department of Internal Medicine, Houston, TX, USA; Weill Cornell – Medical College – Department of Internal Medicine, New York, NY, USA; Houston Methodist Hospital – Department of Internal Medicine, Houston, TX, USA; Weill Cornell – Medical College – Department of Internal Medicine, New York, NY, USA; American University of Beirut – Department of Internal Medicine, Beirut, Lebanon; Harvard Medical School – Department of Internal Medicine, Boston, MA, USA; Houston Methodist Hospital – Department of Internal Medicine, Houston, TX, USA; Weill Cornell – Medical College – Department of Internal Medicine, New York, NY, USA; Houston Methodist Hospital – Department of Internal Medicine, Houston, TX, USA; Weill Cornell – Medical College – Department of Internal Medicine, New York, NY, USA

**Keywords:** pulmonary hypertension, pulmonary thromboendarterectomy, renal failure

## Abstract

The occurrence of renal failure in pulmonary hypertension (PH) is an ominous sign and implies excessive adverse hemodynamic factors. Pharmacologic agents to treat the PH are the mainstay of management, whereas diuretics assist in management of fluid overload. However, when such measures fail, dialysis and ultrafiltration (UF) become necessary to manage progressive azotemia and hypervolemia. Reversal of PH is essential to interrupt this vicious cycle of multisystem failure; otherwise, the need for renal replacement therapy would be permanent.

Chronic thromboembolic pulmonary hypertension (CTEPH) is a unique category of pulmonary hypertension (PH) because substantial improvement of the disease would occur following pulmonary thromboendarterectomy (PTE). PTE is associated with substantial reductions in pulmonary vascular resistance and right heart strain that cannot be achieved by pharmacologic agents alone. Such hemodynamic improvements translate to very favorable effects on renal function.

In this report we describe two patients who had severe PH due to CTEPH. They were refractory to treatment by pharmacologic agents and required initiation of long-term renal replacement therapy (RRT). However, when PTE was performed, not only did they exhibit substantial improvement in the severity of their PH, but both patients were able to get off dialysis. We believe this is the first report of resolution of CTEPH-associated RRT following PTE. This report highlights the importance of screening for CTEPH in individuals with PH, and should encourage treatment of CTEPH by PTE when renal failure is believed to have been induced by PH.

## INTRODUCTION

Acute kidney injury (AKI) is a common complication in patients with advanced pulmonary hypertension (PH) due to the adverse hemodynamic derangements accompanying the disease [1]. Right heart decompensation, increased renal venous pressure and decreased effective perfusion of the kidneys lead to activation of the renin–angiotensin–aldosterone system and induction of the pro-inflammatory cascade, resulting in reduction in the glomerular filtration fraction and oxidative kidney injury [1, 2]. These pathophysiologic derangements in turn lead to progressive azotemia and symptomatic fluid retention.

The primary treatment of PH focuses on short-term reduction of pulmonary artery pressure, and long-term reversal of pulmonary vascular resistance (PVR) [3]. Such treatments are expected to have favorable effects on renal perfusion, but in advanced stages of PH, additional efforts are directed toward management of fluid retention and vascular congestion with the use of diuretics. Eventually, renal replacement therapy (RRT) with ultrafiltration (UF) becomes necessary for those who become refractory to maximal doses of intravenous diuretics [3]. While UF is supposed to assist in reducing central venous pressure and off-load right ventricular strain, it often leads to systemic hypotension, thereby further reducing renal perfusion and aggravating oliguria. Hence, recovery of renal function is unlikely to occur once RRT has been initiated unless significant reduction of PH would be achieved.

In this report, we present two patients with advanced PH due to chronic thromboembolic pulmonary hypertension (CTEPH) who developed acute renal failure requiring prolonged periods of RRT. Their renal failure did not recover following initiation of UF. However, when these patients underwent successful pulmonary thromboendarterectomy (PTE), their renal failure resolved and they were able to get off RRT, and successfully maintained long-term stable kidney function.

## CASE 1 PRESENTATION

A 43-year-old male with antiphospholipid antibody syndrome, type 2 diabetes mellitus, hypertension, hypothyroidism, stage 3 chronic kidney disease (CKD), bilateral pulmonary emboli, on chronic anticoagulation and home oxygen therapy (2 L O_2_ NC), was admitted to our tertiary care hospital in August 2019 with worsening exertional dyspnea, orthopnea, hypoxemia, diuretic resistance and progressive fluid retention. He had several previous hospitalizations since 2016 in various surrounding hospitals for shortness of breath, community-acquired pneumonia and right heart failure. He had already been diagnosed with CTEPH and was advised to undergo PTE, but had previously refused the procedure.

Upon admission, he was afebrile but very dyspneic and hypoxemic; he had significant lower extremity dependent edema and ascites. He had anemia but normal white cell counts. His admission creatinine was 2.32 mg/dL, up from a recent prior baseline of 1.8 mg/dL. His fractional excretion of urea was 19.6% and his urinalysis showed hyaline casts but no proteinuria. His renal ultrasound was normal. His ventilation-perfusion scan showed intermediate to high probability for pulmonary embolism, and a large left lower lobe embolus was suspected. His transthoracic echocardiogram showed a left ventricular ejection fraction (LVEF) >70% but had a severely dilated right ventricle (RV) with reduced RV systolic function. His clinical presentation was that of decompensated right heart failure due to the previously known PH, and his acute kidney failure was judged to be due to prerenal factors.

**Figure 1:**
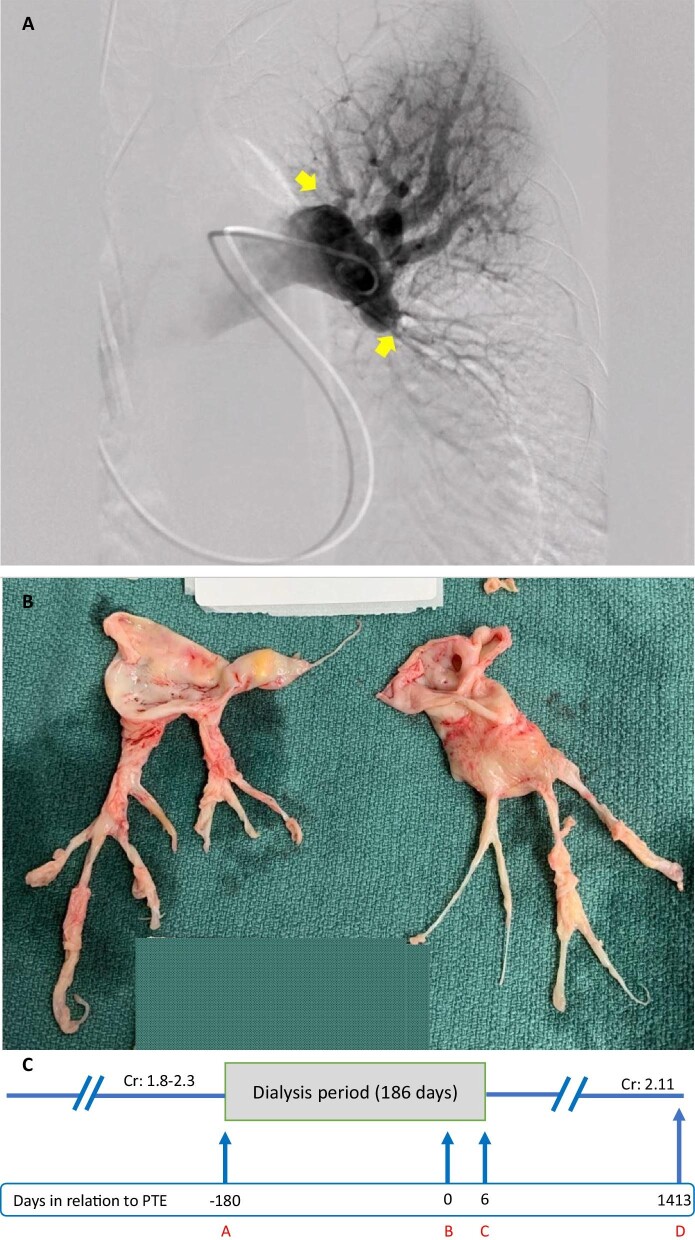
(**A**) Pre-operative left pulmonary artery angiogram of Case 1. Arrows point to occlusions of the pulmonary artery leading to pulmonary hypoperfusion. (**B**) Specimen retrieved from PTE of Case 1. (**C**) A timeline of Case 1. A indicates initiation of dialysis; B indicates day of PTE; C indicates last day of dialysis; D indicates last day of follow-up. Cr: creatinine (mg/dL).

His initial management included supplemental oxygen therapy, high doses of intravenous loop diuretics in combination with thiazide diuretics (bumetanide plus metolazone), anticoagulation and inotropic support. He underwent a right heart catheterization (RHC) which confirmed the presence of severe PH (Table 1). He was given several medications to treat PH and these initially included selexipag (Uptravi) and macitentan (Opsumit), and subsequently riociguat (Adempas) was added. His hospital course was prominent for being refractory to high-dose combination intravenous diuretics. His kidney function continued to deteriorate, and he was started on hemodialysis with UF on 23 August 2019. Once his volume status improved with UF, an attempt was made to discontinue his dialysis, but it failed due to oliguria and rise in azotemia. Thus, plans were undertaken to keep him on dialysis and UF. On 19 September 2019 he underwent a pulmonary arteriogram and a left heart cath. The coronaries were normal, but his pulmonary arteriograms were compatible with CTEPH (Fig. 1A). Cardiothoracic surgery agreed to perform PTE but wanted him to be better conditioned prior to proceeding with surgery. The patient elected to do peritoneal dialysis (PD), so a PD catheter was placed, and he was discharged to rehabilitation on 23 September 2019 to continue dialysis, anticoagulation, PH medications and home oxygen. Another attempt to take him off dialysis in November 2019 failed due to progressive azotemia.

**Table 1:** Invasive hemodynamic parameters determined before and after PTE.

	Pre-PTE		Post-PTE	
	mPAP (mmHg)	PVR (WU)	mPAP (mmHg)	PVR (WU)
Case 1	57	5.8	14	0.7
Case 2	57	6.87	25	

mPAP: mean pulmonary artery pressure, WU: Wood Units.

After several months of rehabilitation and dialysis, he underwent successful open bilateral PTE on 19 February 2020 with removal of organized thromboembolic clots (Fig. 1B). His urine output improved shortly afterwards, and dialysis was permanently stopped on 25 February 2020. He was discharged home in stable condition on 2 March 2020. His PD catheter was removed on 24 June 2020. He had a repeat RHC on 11 February 2021 which showed normal pulmonary artery pressures (Table 1). The chronologic course of the patient's kidney status prior to and following PTE is provided (Fig. 1C). He was continued on combination diuretics that were gradually tapered. His last follow-up with nephrology was on 18 December 2023, when he had a creatinine of 2.11 mg/dL and he was off diuretics.

## CASE 2 PRESENTATION

A 57-year-old woman developed deep vein thrombosis and chronic thromboembolism that led to PH. She was diagnosed with CTEPH in 2010 at age 45 years. She was initially placed on warfarin, but in 2014 she had spontaneous intracranial hemorrhage; thus, warfarin was stopped, and an inferior vena cava filter was placed. She was managed medically with ambrisentan and riociguat, as well as home oxygen. She also developed stage 3 CKD with a serum creatinine of 2 mg/dL. Her condition deteriorated in 2018 due to progressive dyspnea with exertion, orthopnea and progressive lower extremity edema, and required admission to a local hospital on 7 April 2018. Her initial evaluation revealed AKI with rise in creatinine to 4.4 mg/dL. She was started on intravenous bumetanide for management of volume overload, but on 10 April 2018, she was transferred to our tertiary hospital for higher acuity of care.

Upon admission, she was afebrile and hemodynamically stable, but had increased work of breathing, decreased breath sounds at the bases of the lungs, and edema in the lower extremities and abdominal wall. Her initial laboratory workup was significant for hemoglobin of 9.4 g/dL and elevated D dimer of 9.56 µg/mL. Her blood urea nitrogen was 107 mg/dL, creatinine was 5.9 mg/dL and estimated glomerular filtration rate (eGFR) was 7 mL/min/1.73 m^2^. Her admission chest X-ray was significant for enlarged cardiac silhouette, but no pleural effusion was noted. Echocardiography showed normal LV function (LVEF 65%–69%), RV systolic dysfunction, severe tricuspid valve (TV) regurgitation and a moderately large pericardial effusion without signs of tamponade. Her renal ultrasound did not show hydronephrosis. Thus, her clinical presentation was that of decompensated right-sided heart failure due to worsening PH, which in turn led to hemodynamically mediated renal failure.

**Figure 2:**
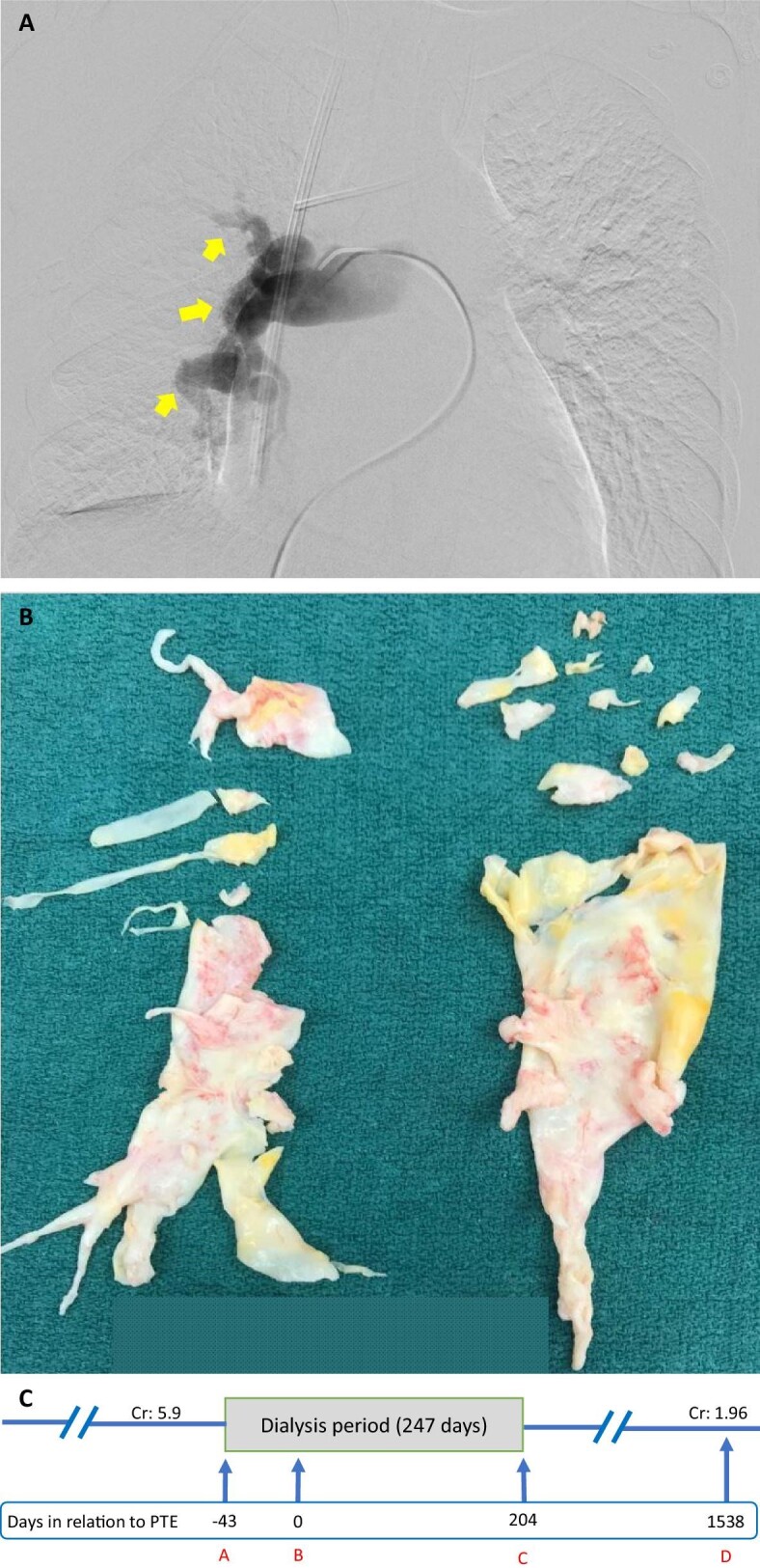
(**A**) Pre-operative right pulmonary artery angiogram of Case 2. Arrows point to occlusions of the pulmonary artery leading to pulmonary hypoperfusion. (**B**) Specimen retrieved from PTE of Case 2. (**C**) A timeline of Case 2. A indicates initiation of dialysis; B indicates day of PTE; C indicates last day of dialysis; D indicates last day of follow-up. Cr, creatinine (mg/dL).

The patient was started on intravenous prostacyclin in addition to her home medications for PH. For her AKI and volume overload, high doses of intravenous furosemide failed to induce acceptable diuresis, so she was started on hemodialysis on 11 April 2018. Initially, she required daily UF for the first 10 consecutive days after admission, then transitioned to three times weekly once euvolemic status was achieved. She underwent a RHC with pulmonary angiograms on 16 May 2018. These demonstrated CTEPH with multifocal occlusions in the pulmonary arteries (Fig. 2A) and elevated central filling pressures with mean pulmonary artery pressures of 51 mmHg bilaterally (Table 1).

The patient underwent PTE (Fig. 2B) and TV repair on 24 May 2018. After the surgery, the patient was transferred to the cardiovascular intensive care unit, where she initially required vasopressors and was placed on continuous RRT. Her pulmonary artery pressures gradually improved (Table 1), but her postoperative course was complicated by cardiac tamponade, for which she underwent emergent pericardiocentesis on 1 June 2018. She was transferred to a regular floor once she became hemodynamically stable on 5 June 2018. She was discharged home on 9 June 2018 on ambrisentan 10 mg daily and intravenous epoprostenol infusion daily for 1 month for her PH. Upon discharge, her creatinine was 5.2 mg/dL and eGFR was 9 mL/min/1.73 m^2^, so she continued dialysis three times weekly. Gradual improvement of her kidney function was noted over the ensuing months, and dialysis was permanently stopped in December 2018.

During her follow-up with her pulmonologist in March 2019, ambrisentan dose was reduced to 5 mg daily. The dialysis catheter was removed on 20 March 2019. The chronologic course of the patient's kidney status prior to and following PTE is provided (Fig. 2C). She was lost to regular follow-up but her last known laboratory tests on 10 August 2022 showed stable kidney function with creatinine of 1.96 mg/dL and eGFR of 29 mL/min/1.73 m^2^.

## DISCUSSION

PH is defined as elevated resting mean pulmonary arterial pressure ≥20 mmHg, which is confirmed by RHC. Based on the underlying etiology, PH is classified into five groups: primary pulmonary arterial hypertension, PH due to left-sided heart disease, PH due to lung disease, CTEPH and PH due to unknown mechanisms [4].

Worsening of renal function, induced by adverse hemodynamic factors, is not unusual in severe cases of precapillary PH, regardless of etiology. These advanced cases are often associated with right heart failure and drop in blood pressure [1]. A vicious cycle of fluid retention, diuretic resistance, increased diuretic requirements and progressive renal failure ensues. Often, it is the refractory fluid retention that forces the initiation of RRT and UF. Thus, RRT in patients with PH is usually initiated at lower creatinine values in comparison with other causes of renal failure [3]. The occurrence of renal failure in PH is a dismal event that has been associated with significant increase in mortality [1, 2]. Additionally, recovery from needing RRT is unlikely to occur unless resolution of PH could be achieved.

CTEPH is a special category of precapillary PH, that is defined as PH with ventilation/perfusion (V/Q) mismatch [5]. It is characterized by occlusion of the large or segmental pulmonary arteries by endothelialized pulmonary thrombi that can be demonstrated on computed tomography pulmonary angiography (CTPA) [2, 5, 6]. CTEPH is treated and potentially cured surgically with PTE, while balloon pulmonary angioplasty and medical therapy are reserved for the inoperable cases [5, 6]. The role of PTE in CTEPH treatment is well-established [5] as it leads to better survival [7] and significant improvement of PH through reduction of the PVR after the surgery [8, 9]. However, there is paucity of data on the role of PTE in improving kidney failure associated with CTEPH [3, 10].

Here, we report two patients with CKD presenting with advanced CTEPH and acute deterioration of their kidney function. In both patients, the renal perfusion was compromised because of adverse hemodynamic factors, and both exhibited extreme diuretic resistance and worsening renal function that forced the initiation of dialysis and UF.

In the first patient, despite initial optimization of central filling pressures by UF and pharmacologic treatment of PH, two attempts were undertaken to get him off dialysis, but both attempts failed due to diuretic resistance, rise in azotemia and progressive fluid retention. In contradistinction, after successful PTE, his renal function improved, his diuretic resistance resolved and he was easily taken off dialysis on day 6 after the procedure. This was a dramatic resolution of renal failure that was temporally linked to the PTE procedure as the definitive treatment of his CTEPH. Upon follow-up 3.5 years later, he continues to have stable renal function with a creatinine of 1.7–2.1 mg/dL, and he is maintaining euvolemia without needing diuretics.

Our second patient illustrates decompensated right-sided heart failure from severe PH complicated by a decompensated TV. The patient required dialysis and UF for 44 days prior to PTE. The PTE procedure was done in conjunction with TV repair. After the procedure, her condition improved, and she gradually regained renal function. The resolution of her renal failure was slower than in our first patient, likely due to multiple postoperative adverse factors that included pericardial tamponade, systemic hypotension and need for postoperative vasopressors. Such events are believed to have induced further renal ischemia and have likely retarded the recovery of renal function in the immediate postoperative period. Nevertheless, she experienced progressive improvement in renal function such that by 6 months after the PTE, she was declared dialysis free.

In summary, we present two patients with severe PH due to CTEPH and prolonged need for RRT. In both patients, not only did PTE achieve significant resolution of their PH symptoms, but it also led to significant improvement of their renal function such that they were both able to get off dialysis and sustain long-term stability of their renal function. To our knowledge, this is the first description of reversal of dialysis-requiring renal failure in patients with CTEPH that was largely achieved through treatment of CTEPH by PTE. This further emphasizes the importance of early distinction of the etiology of PH through screening for CTEPH and timely intervention with PTE that would improve both pulmonary and renal functions.

## Data Availability

Additional supplemental data on file on these 2 patients could be provided on request.

## PATIENT PRIVACY

The privacy of patients in this report was respected by eliminating any personal identifiers, and in compliance with the declaration of Helsinki.

## References

[1] Naranjo M, Lo KB, Mezue K et al. Effects of pulmonary hypertension and right ventricular function in short and long-term kidney function. Curr Cardiol Rev 2018;15:3–11. 10.2174/1573403X14666181008154215 PMC6367698 30306876

[2] Pływaczewska M, Pruszczyk P, Kostrubiec M. Does kidney function matter in pulmonary thromboembolism management? Cardiol J 2022;29:858–65. 10.5603/CJ.a2021.0005 33470418 PMC9550328

[3] Garcia M, Souza R, Caruso P. Renal replacement therapy in patients with acute decompensated pulmonary hypertension admitted to the intensive care unit. Cureus 2022;14:e28792. 36225491 10.7759/cureus.28792 PMC9533720

[4] Mandras SA, Mehta HS, Vaidya A. Pulmonary hypertension: a brief guide for clinicians. Mayo Clin Proc 2020;95:1978–88. 10.1016/j.mayocp.2020.04.039 32861339

[5] Wilkens H, Konstantinides S, Lang IM et al. Chronic thromboembolic pulmonary hypertension (CTEPH): updated recommendations from the Cologne Consensus Conference 2018. Int J Cardiol 2018;272:69–78. 10.1016/j.ijcard.2018.08.079 30195840

[6] Mahmud E, Madani MM, Kim NH et al. Chronic thromboembolic pulmonary hypertension: evolving therapeutic approaches for operable and inoperable disease. J Am Coll Cardiol 2018;71:2468–86. 10.1016/j.jacc.2018.04.009 29793636

[7] Cannon JE, Su L, Kiely DG et al. Dynamic risk stratification of patient long-term outcome after pulmonary endarterectomy: results from the United Kingdom national cohort. Circulation 2016;133:1761–71. 10.1161/CIRCULATIONAHA.115.019470 27052413 PMC5860739

[8] Jenkins D. Pulmonary endarterectomy: the potentially curative treatment for patients with chronic thromboembolic pulmonary hypertension. Eur Respir Rev 2015;24:263–71. 10.1183/16000617.00000815 26028638 PMC9487822

[9] Skoro-Sajer N, Marta G, Gerges C et al. Surgical specimens, haemodynamics and long-term outcomes after pulmonary endarterectomy. Thorax 2014;69:116–22. 10.1136/thoraxjnl-2013-203746 24052543 PMC3913220

[10] Sztrymf B, Prat D, Jacobs FM et al. Renal replacement therapy in patients with severe precapillary pulmonary hypertension with acute right heart failure. Respiration 2013;85:464–70. 10.1159/000339346 22906846

